# Genome-wide single-nucleotide polymorphism analysis revealed SUFU suppression of acute graft-versus-host disease through downregulation of HLA-DR expression in recipient dendritic cells

**DOI:** 10.1038/srep11098

**Published:** 2015-06-11

**Authors:** Rafijul Bari, Christine Hartford, Wing Keung Chan, Queenie Vong, Ying Li, Kwan Gan, Yinmei Zhou, Cheng Cheng, Guolian Kang, Sheila Shurtleff, Victoria Turner, Ching-Hon Pui, James R. Downing, Wing Leung

**Affiliations:** 1 Department of Bone Marrow Transplantation and Cellular Therapy, St. Jude Children’s Research Hospital, Memphis, TN; 2 Department of Biostatistics, St. Jude Children’s Research Hospital, Memphis, TN; 3 Department of Pathology, St. Jude Children’s Research Hospital, Memphis, TN; 4 Department of Oncology, St. Jude Children’s Research Hospital, Memphis, TN; 5 Department of Pediatrics, University of Tennessee Health Science Center, Memphis, Tennessee, USA

## Abstract

Graft-versus-host disease (GVHD) is a major cause of morbidity and mortality after allogeneic hematopoietic stem cell transplantation (HSCT). To identify recipient risk factors, a genome-wide study was performed including 481,820 single-nucleotide polymorphisms (SNPs). Two GVHD susceptibility loci (rs17114803 and rs17114808) within the *SUFU* gene were identified in the discovery cohort (p = 2.85 × 10^−5^). The incidence of acute GVHD among patients homozygous for CC at *SUFU* rs17114808 was 69%, which was significantly higher than the 8% rate observed in CT heterozygous patients (p = 0.0002). In an independent validation cohort of 100 patients, 50% of the patients with the CC genotype developed GVHD compared to 8% of the patients with either CT or TT genotype (p = 0.01). In comparison to CC dendritic cells, those from CT expressed higher levels of *SUFU* mRNA and protein, had lower levels of surface HLA-DR, and induced less allogeneic mixed leukocyte response (MLR). Ectopic expression of *SUFU* in THP-1 derived DCs reduced HLA-DR expression and suppressed MLR, whereas silencing of *SUFU* enhanced HLA-DR expression and increased MLR. Thus our findings provide novel evidence that recipient *SUFU* germline polymorphism is associated with acute GVHD and is a novel molecular target for GVHD prevention and treatment.

Hematopoietic stem cell transplantation (HSCT) is used to treat a variety of malignant and non-malignant diseases. Successful allogeneic HSCT involves intensive immunosuppression of the recipient, followed by infusion of the donor stem cell graft. In addition to hematopoietic stem cells, the graft also contains CD4^+^ and CD8^+^ αβ T-cells. One of the main benefits of allogeneic HSCT is the alloreactivity of the donor T-lymphocytes toward recipient malignant cells, leading to the beneficial graft-versus-malignancy effect^1^. However, this non-specific alloreactivity may also direct toward normal tissues in the recipient, resulting in graft-versus-host disease (GVHD)^2^,^3^.

Although our understanding of the pathophysiology of GVHD improved substantially, little progress has been made in the treatment of GVHD since the introduction of calcineurin-inhibitor-based regimens in the 1980s^4^. Many factors, related to both the donor and the recipient, have been identified as potential risk factors for the development of GVHD^5^. The most important risk factor is the genetic disparity between the donor and recipient in human leukocyte antigen (HLA)^6^. The frequency of acute GVHD is directly related to the degree of HLA mismatch between the donor and recipient^7^. Furthermore, about 40% of recipients of HLA-identical grafts experience acute GVHD triggered by disparity in minor antigens^8^. Relatively little is known about non-HLA genetic factors in the recipient that may contribute to the development of GVHD^9^,^10^. Identifying such factors is useful because it will allow development of novel molecular targeted therapy, improved risk stratification, and individualized GVHD prophylaxis and treatment. Patients at low risk for the development of acute GVHD may have immunosuppression decreased to safely allow a stronger graft-versus-leukemia effect, while those at high risk for GVHD may require a more intensive or prolonged immunosuppression regimen to prevent GVHD mortality.

Single-nucleotide polymorphism (SNP) is a common form of natural genetic variation. Genome-wide analyses of germline SNPs have identified inherited polymorphisms associated with treatment response and treatment-related adverse effects in patients with leukemia^11^,^12^,^13^,^14^. The candidate gene approach has identified polymorphisms of a number of genes associated with a variety of HSCT-related outcomes, including infection^15^,^16^,^17^, GVHD^18^,^19^, liver toxicities^20^,^21^, and relapse risk^22^,^23^. However, there are very few genome-wide studies among HSCT patients^24^,^25^,^26^, and no study has focused on the pediatric population, which accounts for one-third of allogeneic HSCT recipients worldwide. In this study, we investigated the role of recipient germline SNPs in the development of acute GVHD in a group of pediatric patients who received allogeneic HSCT at a single institution. We identified two SNPs in *suppressor of fused (SUFU)* that were associated with acute GVHD and elucidated the mechanisms of action.

## Results

### Genome-wide screening and validation of SNPs associated with acute GVHD

**Figure 1:**
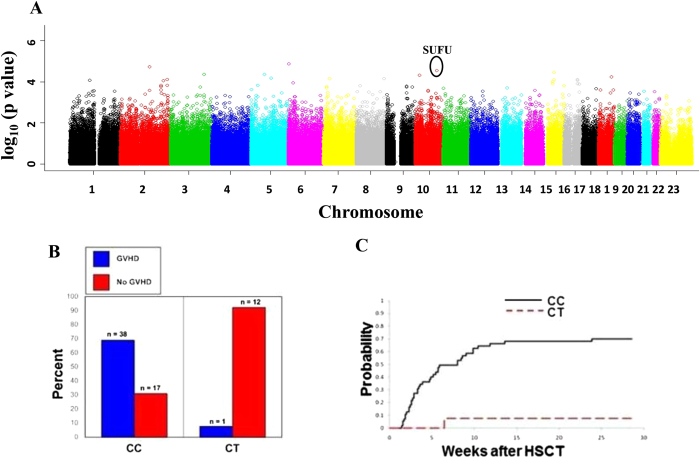
Genome-wide screening of SNPs associated with acute GVHD in patients who underwent HSCT. **A**, Manhattan plot of p-values from genome-wide association analyses. The horizontal axis indicates each SNP’s chromosomal physical location, while the vertical axis indicates the degree of SNP association with acute GVHD (-log_10_(p-value)). **B**, Percent and number (n) of subjects with and without acute GVHD stratified by rs17114808 SNP genotype in the discovery cohort. **C**, Cumulative incidence of acute GVHD in the discovery cohort: 69% among CC, 8% among CT (p = 0.0002). CC indicates patients homozygous for *SUFU* allele with cytosine at rs17114808 position; CT indicates heterozygous with cytosine and thymine at the same position.

Of the 68 patients in the discovery cohort, 39 (57%) experienced acute GVHD as defined by standard criteria^27^. After quality control filters were applied, 305,830 SNPs were evaluated in 68 patients in the discovery cohort. By the information profile selection criteria, 16 of the 305,830 SNPs were chosen based on the p-value of the hybrid-permutation method as being significantly associated with acute GVHD. A Manhattan plot of the chromosomal locations is shown in Fig. 1A and the corresponding Q-Q plot is shown in supplementary Figure 1.

Among the 16 top SNPs associated with acute GVHD from the genome wide analysis, two were in *SUFU*: a coding synonymous SNP at rs17114803 (p = 2.85 × 10^−5^) and another SNP in the 3’ untranslated region (3’ UTR), rs17114808 (p = 2.85 × 10^−5^) as demonstrated in Fig. 1A. These SNPs are in complete linkage disequilibrium and are located on chromosome 10. Among the 68 patients in the discovery cohort, 55 (81%) were homozygous for the major allele with cytosine at rs17114808 (CC), no patient was homozygous for the minor allele thymine at this position (TT), and 13 (19%) were heterozygous with cytosine and thymine at the same position (CT). There were no clinical or demographic features that were statistically different among the different genotype groups (Table 1). The cumulative incidence of acute GVHD was 69% among patients homozygous for the C allele, and only 8% among those who were heterozygous (p = 0.0002) (Fig. 1B,C), suggesting a protective effect by the T allele. Furthermore, a full spectrum of GVHD severity was observed in the CC group, whereas only grade I GVHD was seen in the single case among CT recipients (Table 1). There were no clinical or demographic factors that were statistically different in patients with and without acute GVHD (Table 2). Therefore, of all factors investigated, *SUFU* SNP genotype was the only factor significantly associated with acute GVHD in the discovery cohort.

**Figure 2:**
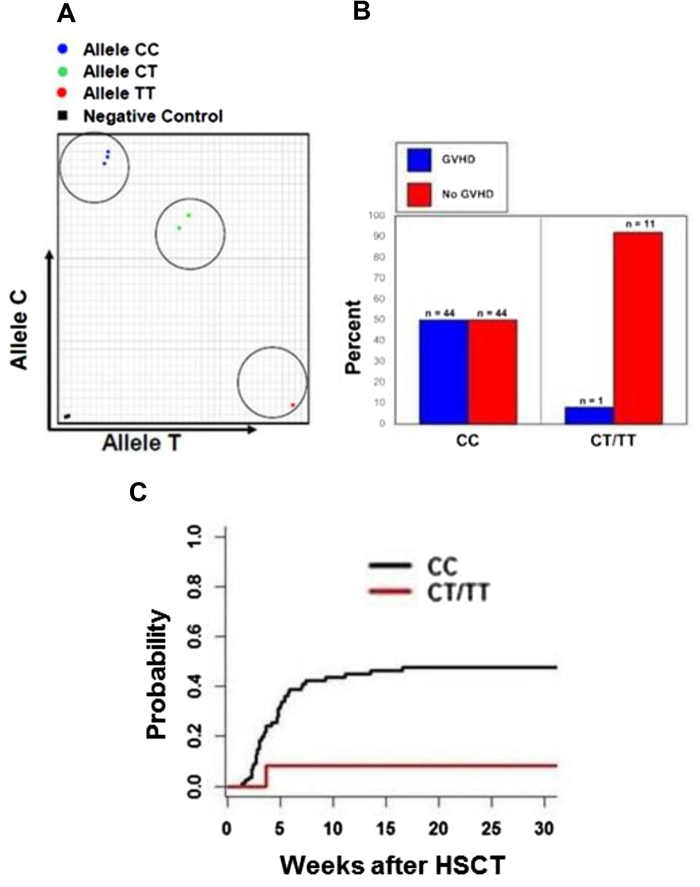
Validation of SUFU SNPs in association with acute GVHD. **A**, A SNP assay was developed that can distinguish different *SUFU* genotypes in the validation cohort. A representative typing output is shown. **B**, Percent and number (n) of subjects with and without acute GVHD stratified by rs17114808 SNP genotype in the validation cohort. **C**, Cumulative incidence of acute GVHD in the validation cohort: 50% among CC, 8.3% among CT/TT (p = 0.01). CC indicates patients homozygous for *SUFU* allele with cytosine at rs17114808 position; CT indicates heterozygous with cytosine and thymine at the same position; and TT indicates homozygosity with thymine in that position.

To validate our discovery cohort results, we developed a PCR-based SNP assay that can detect the presence of different SUFU alleles (Fig. 2A). We sequenced the PCR products from each group (CC, CT, and TT) and confirmed the accuracy of the assay (supplemental Figure 2). Using the SNP assay, we genotyped another 100 patients who had undergone HSCT at St. Jude as an independent validation cohort. Among these 100 patients, 88% were CC homozygous, 3% were TT homozygous and 9% were CT. The cumulative incidence of acute GVHD was 50% among CC homozygous, but was only 8.3% among those who were heterozygous or homozygous for the T allele (p = 0.01) (Fig. 2B,C); again, only a single case of grade I GVHD was observed among recipients with T allele, but more severe GVHD was seen in CC homozygous subjects (Table 1). Similar to the discovery cohort, there were no significant differences in any patient demographic or transplant-related characteristics between the allelic groups in the validation cohort (Table 1). Table 2 demonstrates the demographic and transplant characteristics according to acute GVHD in the validation cohort. Besides *SUFU* genotype, age at HSCT, CNI use and MMF use were significantly different between those with and without acute GVHD. Univariate logistical regression analysis confirmed the associations between *SUFU* SNP genotype (p = 0.024), age at HSCT (p = 0.032), CNI use (p = 0.0019) and MMF use (p = 0.0025) and acute GVHD. Multivariate analysis showed that *SUFU* SNP genotype (p = 0.025) was still statistically associated with acute GVHD after adjusting for age at HSCT, CNI and MMF, suggesting that *SUFU* SNP is an independent predictor of acute GVHD. The same risk factors for acute GVHD (age at HSCT, p = 0.035; CNI, p = 0.0034; and MMF, p = 0.0036) were identified by time-to-event analysis using Fine and Gray’s cumulative incidence model. Multivariate analysis of cumulative incidences showed that *SUFU* SNP was still statistically associated with acute GVHD (p = 0.048) after adjusting for age at HSCT, CNI and MMF.

### *SUFU* SNPs correlated with mRNA and protein level in PBMCs

**Figure 3:**
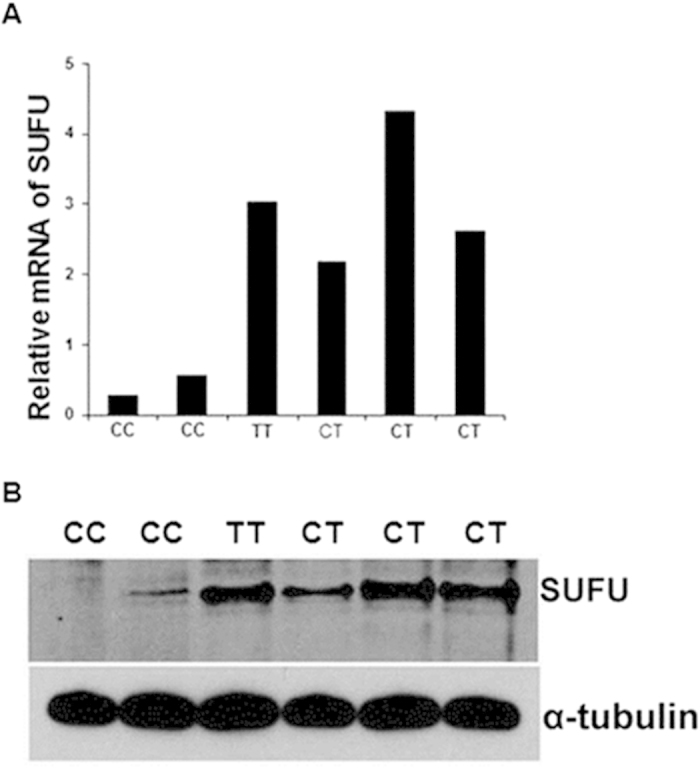
*SUFU* polymorphism in healthy population. **A**, Relative abundance of SUFU mRNA in PBMCs from six representative healthy individuals with homozygous (CC or TT) or heterozygous (CT) alleles. Data are normalized to GAPDH mRNA levels and are presented as fold change relative to the expression of GAPDH. **B**, SUFU protein production in PBMCs from the same six healthy individuals was determined by Western blot. α-tubulin was used as a loading control.

*SUFU* is not known to play a role in human immunology, and the biology of *SUFU* SNPs has not been elucidated. To further investigate the effect of *SUFU* allelic polymorphism, we genotyped 30 healthy subjects and found that 25 were CC (83.33%), 4 were CT (13.33%), and 1 was TT (3.33%). This distribution was similar to that of the HSCT patients in our study. Because *SUFU* rs17114808 SNP is located in the 3’ UTR, we hypothesized that the SNP did not affect protein structure and function but might affect its abundance through translational control, degradation of mRNA or subcellular localization^28^,^29^,^30^,^31^. We quantified the *SUFU* transcripts by RT-qPCR using *SUFU*-specific QuantiTect RT-PCR primers and found less *SUFU* transcript in PBMCs from individuals with the CC genotype than in those with CT or TT genotypes (Fig. 3A). Similarly, PBMCs from individuals who were CC homozygous produced much less SUFU protein than those from people who were heterozygous (CT) or homozygous (TT) for the minor *SUFU* allele (Fig. 3B).

### SUFU suppresses HLA-DR expression in blood DCs and reduces their ability to induce allogeneic T-cell proliferation

**Figure 4:**
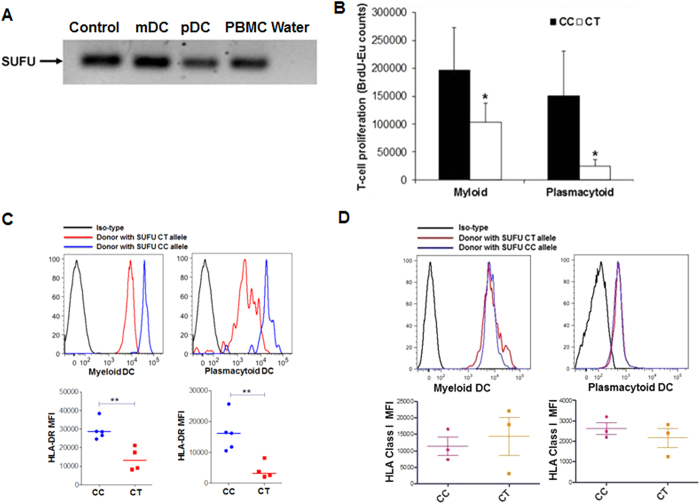
*SUFU* suppresses allogeneic T-cell proliferation by reducing HLA-DR expression in dendritic cells. **A**, Expression of *SUFU* transcripts in mDCs, pDCs, and PBMCs. Plasmid DNA of *SUFU* used as positive control and water as a negative control. **B**, DCs were isolated from *SUFU*-CC– and *SUFU*-CT–positive donors and were used for allogeneic MLR assays. Shown are data from 3 independent experiments. **C**, mDCs (upper left) and pDCs (upper right) were isolated from blood of healthy volunteers with different groups of *SUFU* alleles, and their HLA-DR expression was determined by flow cytometry. Mean fluorescence intensity (MFI) of HLA-DR expression in mDCs (**C**, bottom left) and pDCs (**C**, bottom right) from 4 to 5 volunteers of each SUFU allelic group is plotted. **D**, Representative mean fluorescence intensity (MFI) of MHC class I expression on myeloid (upper left) and plasmacytoid DC (upper right) from individuals with different SUFU allele are shown. Average MFI of MHC class I expression from 3 individuals from each group of SUFU alleles are shown at the bottom. mDC indicates myeloid dendritic cells; pDC indicates plasmacytoid dendritic cells; PBMC indicates peripheral blood mononuclear cells. * indicates p-value <0.05, ** indicates p-value <0.01.

Since GVHD involves stimulation and activation of donor T-cells by recipient antigen-presenting cells (APCs), we hypothesized that SUFU might affect antigen presentation by recipient APCs. Based on our laboratory findings that the T allele was associated with more *SUFU* mRNA and protein in PBMCs and on our clinical observation that CT/TT recipients had less GVHD than CC recipients, we hypothesized that SUFU inhibited GVHD by reducing antigen presentation by recipient APCs. To test our hypotheses, we first purified mDCs and pDCs from healthy individuals to confirm the presence of *SUFU* transcripts in the DC populations using *SUFU*-specific RT-PCR primers (Fig. 4A). We then analyzed the capability of induction of alloreactivity by mDCs and pDCs from individuals having different *SUFU* alleles using MLR assay. DCs from individuals having the *SUFU* T allele induced significantly less allogeneic T-cell proliferation than DCs from individuals with the CC genotype that produced less SUFU protein (Fig. 4B). Furthermore, DCs from CT heterozygous individuals had significantly lower expression of HLA-DR than those from CC homozygous individuals (Fig. 4C). In contrast, there were no significant differences of HLA class I expression in both myeloid and plasmacytoid DCs from individual with different SUFU alleles (Fig. 4D). These findings support the hypothesis that DCs with the T allele expressed more SUFU, which in turn suppressed HLA-DR expression specifically and reduced GVHD potential.

### Overexpression and silencing of SUFU in THP-1–derived DCs changed their HLA-DR expression and ability to induce allogeneic T-cell proliferation

**Figure 5:**
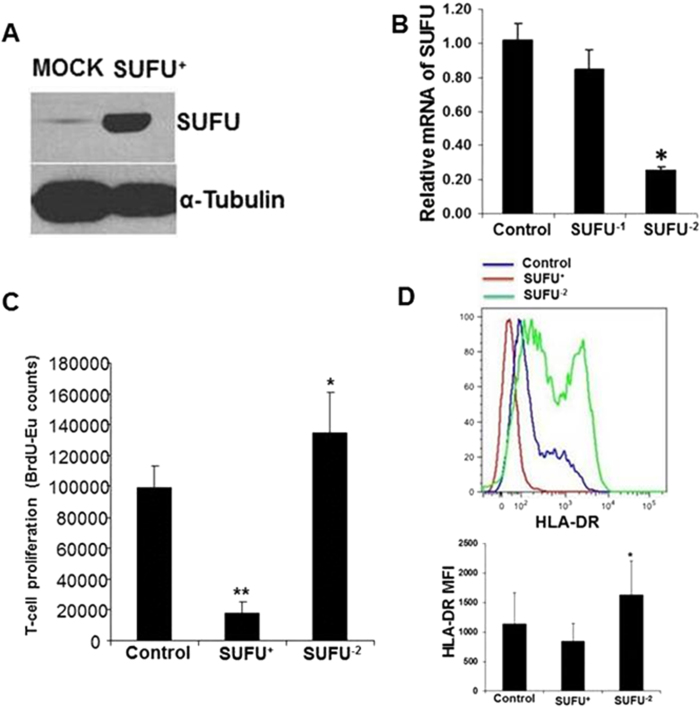
Overexpression of *SUFU* reduced but silencing increased allogeneic T-cell proliferation in MLR using THP-1 cells induced into APCs. *SUFU* was either ectopically expressed in myeloid cell line THP-1 (**A**) or silenced in THP-1 cells by siRNA (**B**). **C**, *SUFU* overexpressed and silenced THP-1 cells were then induced into APCs and used for MLR. **D**, Surface expression of HLA-DR (upper) and MFI (bottom) are shown. Mock indicates THP-1 cells without genetic manipulation; SUFU^+^ indicates ectopically SUFU-expressing THP-1 cells; SUFU^−1^ and SUFU^−2^ indicate two separate siRNAs used to silence SUFU in THP-1 cells; * indicates p-value <0.05, ** indicates p-value <0.01.

To confirm the direct involvement of SUFU in HLA-DR expression and alloreactivity, we used a myeloid cell line, THP-1, that can be induced into APCs by a combination of cytokines^32^,^33^. After culturing THP-1 cells in APC induction conditions, we found higher expression of DC-associated markers than in cells grown in normal growth medium (supplemental Figure 3). We then ectopically expressed (*SUFU*^+^) or silenced *SUFU* expression (*SUFU*^**−**^) in the THP-1 cells (Fig. 5A,B) and used them for allogeneic MLR assay. Overexpression of SUFU inhibited allogeneic T-cell proliferation, whereas silencing of SUFU increased it (Fig. 5C). This cell-line model confirmed our earlier observation that SUFU affected allogeneic T-cell proliferation induced by healthy donor DCs. Furthermore, we found that silencing SUFU increased specifically the expression of HLA-DR, whereas overexpression reduced it (Fig. 5D), but there was no change in expression of other DC markers such as CD40, CD80, CD83, or CD86 (supplemental Figure 4). These findings suggest that SUFU regulates the level of HLA-DR expression in DCs and thus alters allogeneic MLRs. By contrast, there were no differences in HLA-Class I or HLA-DR expression among THP-1, THP-SUFU^+^ and THP-SUFU^−^ cell lines cultured in normal growth medium without differentiating the cells into DCs (supplementary figure 5A and 5B).

## Discussion

In this study, a SNP in the *SUFU* gene (rs17114808) was found to be associated with the incidence of acute GVHD in two independent cohorts of pediatric and young adult patients who underwent allogeneic HSCT for a variety of underlying diagnoses and utilizing various transplant approaches. Transplant recipients who were *SUFU* CC homozygous were more susceptible to acute GVHD than recipients who had CT or TT genotypes. Remarkably, the acute GVHD in recipients with T allele was at most grade I, whereas more severe GVHD was observed in the CC group. The SNP is located in the 3’ UTR of the *SUFU* gene and regulates the quantity of transcript and total protein production. DCs from individuals who are CC homozygous have less SUFU protein, higher level of HLA-DR expression, and stronger potential to induce alloreactive T cell response.

SUFU is a known repressor of the sonic hedgehog (Shh) signaling pathway. Shh acts as a classical morphogen during embryonic development, regulating the pattern of formation in the nervous, respiratory, and intestinal systems^34^,^35^,^36^,^37^. Postnatally, Shh pathways regulate tumorigenesis by controlling gene transcription and autophagy to maintain normal cell homeostasis^38^,^39^. Shh signaling activity is governed by the balance of Gli activators and repressors^40^. SUFU is the core intracellular negative regulator of Shh signaling, interacting directly with Gli to control protein processing, stabilization, and subcellular distribution^41^,^42^,^43^,^44^,^45^,^46^. Although the correlation between SUFU and GVHD was unknown, Pawei Zerr *et al.*^47^ recently reported that Shh signaling is activated in human and murine chronic GVHD. They found that pharmacologic inhibition of Smo, an important co-receptor of the Shh signaling pathway, is effective for prevention and treatment of chronic GVHD. Moreover, Varas *et al.*^48^ reported that Shh is anti-apoptotic in thymic DCs, and blockade of Shh signaling by cyclopamine abrogates the upregulation of HLA-DR expression in DCs induced by CD40 ligands; although the precise molecular mechanism was not elucidated. Here, we found that SUFU is capable of directly reducing HLA-DR expression in both mDCs and pDCs. It is known that GVHD-associated T helper cell responses specific for minor histocompatibility antigens are mainly restricted by HLA-DR molecules 49. HLA-DR–silenced APCs lose their ability to induce proliferation and activation of allogeneic T-cells^50^, which is essential for the development of GVHD. DCs from CT individuals have higher amount of SUFU, less HLA-DR expression, and reduced capacity to stimulate allogeneic T-cell proliferation as compared to CC homozygous.

The primary strength of our study is that this is the first high-density genome-wide SNP study in HSCT recipients rather than donors, identifying the most statistically significant germline molecular determinant for GVHD development, and the only study to include functional validation. In addition to revealing a novel molecular marker for GVHD, the laboratory investigations showed the biological effect of the *SUFU* SNP, thereby providing the pathophysiologic mechanism for the effect of this SNP on GVHD risk. Another strength of this study is the development of a novel and simple assay for *SUFU* allele typing, which was then used to genotype the SNP in an independent cohort of patients. The SNP assay is expedient for testing patients undergoing HSCT and therefore has the potential to be useful in prognostication and in GVHD clinical management. The primary limitation of this study is the small number of patients and the potential for false positive results from the genome-wide scan. We used the hybrid-permutation method to limit false positivity and information profile method to estimate the false discovery rate. In addition, the association between the SNP genotype and the incidence of acute GVHD was validated in an independent cohort, providing additional support. The biological mechanism elucidated for the effect of the SUFU SNP on GVHD further strengthens the validity of our conclusions. Future studies should assemble a larger cohort of HSCT patients across all age groups to further examine the relationship between *SUFU* alleles and the risk of GVHD in various HSCT settings.

In summary, we identified SUFU as a novel molecular determinant for acute GVHD using genome-wide analysis. SUFU may serve as a useful biomarker for individualized treatment and preventive approaches for GVHD in patients undergoing HSCT. For instance, patients who are CC homozygous may benefit from more intensive GVHD prophylaxis, while patients with CT or TT genotypes may receive HSCT from a less-than-perfectly matched donor. Moreover, *SUFU* and the Shh signaling pathway could be novel targets for prevention of GVHD.

## Methods

### Patients, donors, and transplant regimen

For the discovery cohort, germline samples were available for 38 patients with ALL and 30 with AML who underwent HSCT at St. Jude Children’s Research Hospital between 1995 and 2007. For the validation cohort, samples were available from an additional 100 patients who also underwent HSCT at St. Jude during the same time period. All patients in the discovery cohort were treated with a myeloablative conditioning regimen which included total body irradiation (doses 1200-1400 cGY) in the majority of patients. The validation cohort included patients with both malignant and nonmalignant diseases and a wide variety of treatment regimens, including some reduced intensity regimens, thus allowing the evaluation of the generalizability of the *SUFU* SNP effects in various transplant settings.

### Genome-wide screening and statistical analysis

Germline DNA was extracted from patient samples obtained before HSCT and the discovery cohort was genotyped using the Affymetrix GeneChip Human Mapping 500 K set or the Affymetrix Genome-wide Human SNP Array 6.0 (Affymetrix) as previously described^12^,^51^,^52^. Together, genotypes at 481,820 SNPs were generated common on these two arrays. SNPs with a minor allele frequency less than 5% or call rates less than 95% were excluded from the analyses. Each SNP was coded as 0 (AA), 1 (AB), or 2 (BB). The complication of acute GVHD was defined as any stage of acute GVHD in any organ (skin, liver, or gastrointestinal) and coded as 1 (yes) or 0 (no).

To test the associations between each SNP in the genome-wide scan and acute GVHD, the Spearman rank correlation test was used^10^,^53^. Due to the small sample size and to provide effective control of the type I error rate, p-values were obtained by a hybrid-permutation method with 2000 permutations^12^. The profile information threshold method^54^ was used to select SNPs significantly associated with acute GVHD and estimate the false discovery rate for the corresponding p-value cutoff.

For both the discovery and validation cohorts, continuous variables for patient and transplant characteristics between different SNP groups, as well as between those with and without acute GVHD, were compared using the Wilcoxon rank-sum test. Categorical variables were compared using the Pearson’s Chi-square test or Fisher’s exact test. The survival probabilities after HSCT were estimated using Kaplan-Meier method and compared using the Mantel-Haenszel statistic^55^. Cumulative incidences of GVHD were estimated using the methods of Kalbfleisch and Prentice^56^ and compared using the methods of Gray^57^, with adjustment for competing risk of death. Univariate and multivariate Fine and Gray’s and logistic regression models were used to test associations between clinical and genetic factors and acute GVHD. Factors with p-values less than 0.1 were included in the multivariate analysis. We also performed backward stepwise regression for Fine and Gray’s regression model based on BIC to select the final model using R package crrstep http://cran.r-project.org/web/packages/crrstep/index.html.

### Cell line, culture, isolation of blood dendritic cells, and generation of THP-1–derived dendritic cells

Peripheral blood mononuclear cells (PBMCs) were obtained from healthy volunteers with informed consent under a protocol approved by our institutional review board in accordance with the Declaration of Helsinki. Peripheral blood myeloid dendritic cells (mDCs) and plasmacytoid dendritic cells (pDCs) were isolated from PBMCs cultured overnight using the Myeloid or Plasmacytoid Dendritic Cell Isolation Kit following the manufacturer’s instructions (Miltenyi Biotech)^58^. Myeloid cell line THP-1 was purchased from American Type Culture Collection. Cells were cultured in RPMI 1640 medium containing 10% fetal bovine serum, 100 U/mL penicillin, and 100 mg/mL streptomycin (Invitrogen Life Technologies) and were maintained at 37 °C in a humidified atmosphere with 5% CO_2_. THP-1–derived dendritic cells (DCs) were generated as previously described^32^. Briefly, THP-1 cells at a density of 1 × 10^5^ per well were cultured in the presence of granulocyte-macrophage colony-stimulating factor (2500 U/mL; Miltenyi) and interleukin-4 (250 U/mL; R&D Systems Inc.) for 5 days at 37 °C under 5% CO_2_. On day 3, 90% of the medium was replaced with fresh medium and cytokines. On day 5, DC growth medium was replaced with medium containing maturation cytokines, including interleukin-1β (10 ng/mL), interleukin-6 (10 ng/mL), tumor necrosis factor α (10 ng/mL), and prostaglandin E_2_ (1 μg/mL). DCs were then harvested on day 7 and washed for further assays.

### SUFU SNP assay

To detect the presence of various alleles of *SUFU*, a single-nucleotide mismatch detection assay was developed as described previously^59^. Briefly, primers for the assay were designed in such a way that they amplified all alleles of the *SUFU* gene as well as the amplicon containing the polymorphic region of interest. The forward primer was 5′-CCCCTTTCCTGCCTTCTTACC-3′ and the reverse primer was 5′-TCATGACTTTGCTTTGAAGAGGTGTA-3′. The probe for *SUFU* alleles with a thymine at position rs17114808 was 6Fam ATGGGACTGTTATAATACT-MGBNFQ and for those with a cytosine at the same position was VIC TGGGACTGTTACAATACT-MGBNFQ. Each assay reaction mix contained a 250 nM probe concentration and 100 ng of genomic DNA in 1 × TaqMan genotyping master mix (Applied Biosystems). The assay was performed on an HT7900 Sequence Detection System (Applied Biosystems) following the allelic discrimination assay protocol provided by the manufacturer.

### RT-qPCR and siRNA gene silencing

Total RNA was extracted from PBMCs or cell lines using RNA extraction kits (Qiagen). cDNA was generated from the total RNA using SuperScript Reverse Transcriptase (Invitrogen). *SUFU* transcript was quantified using a *SUFU*-specific QuantiTect Primer Assay (Qiagen) according to the manufacturer’s instructions. *SUFU* expression in THP-1 cells was silenced using siRNA (Open Biosystems) following the manufacturer’s instructions.

### *SUFU* cloning, expression, and Western blot

Total RNA was extracted from PBMCs using RNA extraction kits (Qiagen). cDNA was generated from the total RNA using SuperScript Reverse Transcriptase (Invitrogen), and *SUFU* was amplified by PCR and cloned into mammalian expression vector pcDNA3 (Invitrogen). The identity of *SUFU* was confirmed by sequencing. THP-1 cells were transfected with pcDNA3 vector containing *SUFU* by electroporation (Gene Pulser II; Bio-Rad). Stable cell lines were generated by selection in Geneticin (Invitrogen).

For Western blots, the cells were lysed by adding lysis buffer (1% Triton X-100, 150 mM NaCl, and 50 mM Tris [hydroxymethyl] aminomethane-HCl, pH 7.4). Lysed cells were centrifuged, and supernatants were electrophoresed on 4% to 12% NuPAGE Bis-Tris gel (Invitrogen). Separated proteins were blotted with SUFU-specific antibody (Open Biosystems) using a Western blotting protocol as described previously^60^. Pico-enhanced Chemiluminescent Substrate (Thermo Scientific) was used to detect overexpressed SUFU protein in THP-1 cells. The membrane was stripped with Restore Plus Western Blot Stripping Buffer (Thermo Scientific) and reblotted with anti-tubulin antibody (Sigma-Aldrich) as a loading control.

### Mixed leukocyte response (MLR)

The THP-1–derived DCs, or mDCs and pDCs, were irradiated at 30 Gy and co-cultured at a ratio of 1:10 with 1 × 10^5^ allogeneic responder CD3^+^ T-cells from a single donor in flat-bottom 96-well microtiter plates. Cell proliferation was quantified using the DELFIA Cell Proliferation kit (PerkinElmer) following the manufacturer’s instructions. Briefly, bromodeoxyuridine (BrdU) was added into the wells 16 h before the end of a 5-day culture. The next day, cells were fixed and spun down. The supernatant was discarded, anti-BrdU-Eu was added, and the fluorescence was measured using a Wallac Victor 2 Counter Plate Reader (both from PerkinElmer Life and Analytical Sciences).

### Flow cytometry

The following antibodies were purchased from commercial suppliers and used for phenotypic analysis: FITC-conjugated anti-BDCA-1, anti-BDCA-2, anti-CD45, anti-CD80, anti-CD83, anti-CD69, anti-CD45RA, and anti-CD11b; PE-conjugated anti-BDCA-3, anti-BDCA-4, anti-HLA-ABC, anti-CD19, anti-CD20, anti-CD25, anti-CD40, anti-CD86, anti-CCR7, anti-HLA-ABC, and anti-HLA-DR; ECD-conjugated anti-HLA-DR, anti-CD3, and anti-CD62L; APC-conjugated anti-CD11c, anti-CD123, and anti-CD56; APC-Cy7–conjugated anti-CD14, anti-CD3, anti-CD4, and anti-CD19. Flow cytometric analyses were conducted with LSRII (BD Bioscience), and the data were analyzed with FlowJo 8.8.6 (Tree Star).

## Additional Information

**How to cite this article**: Bari, R. *et al.* Genome-wide single-nucleotide polymorphism analysis revealed SUFU suppression of acute graft-versus-host disease through downregulation of HLA-DR expression in recipient dendritic cells. *Sci. Rep.* **5**, 11098; doi: 10.1038/srep11098 (2015).

## Supplementary Material

Supplementary Information

## Figures and Tables

**Table 1:**
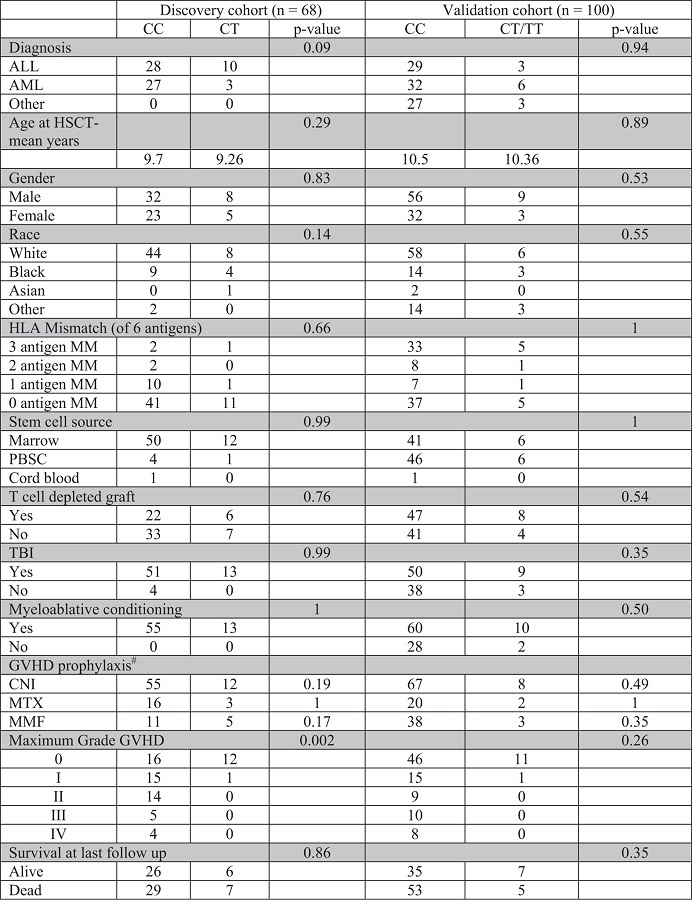
Patient and transplant characteristics of the discovery and validation cohorts.

**Table 2:**
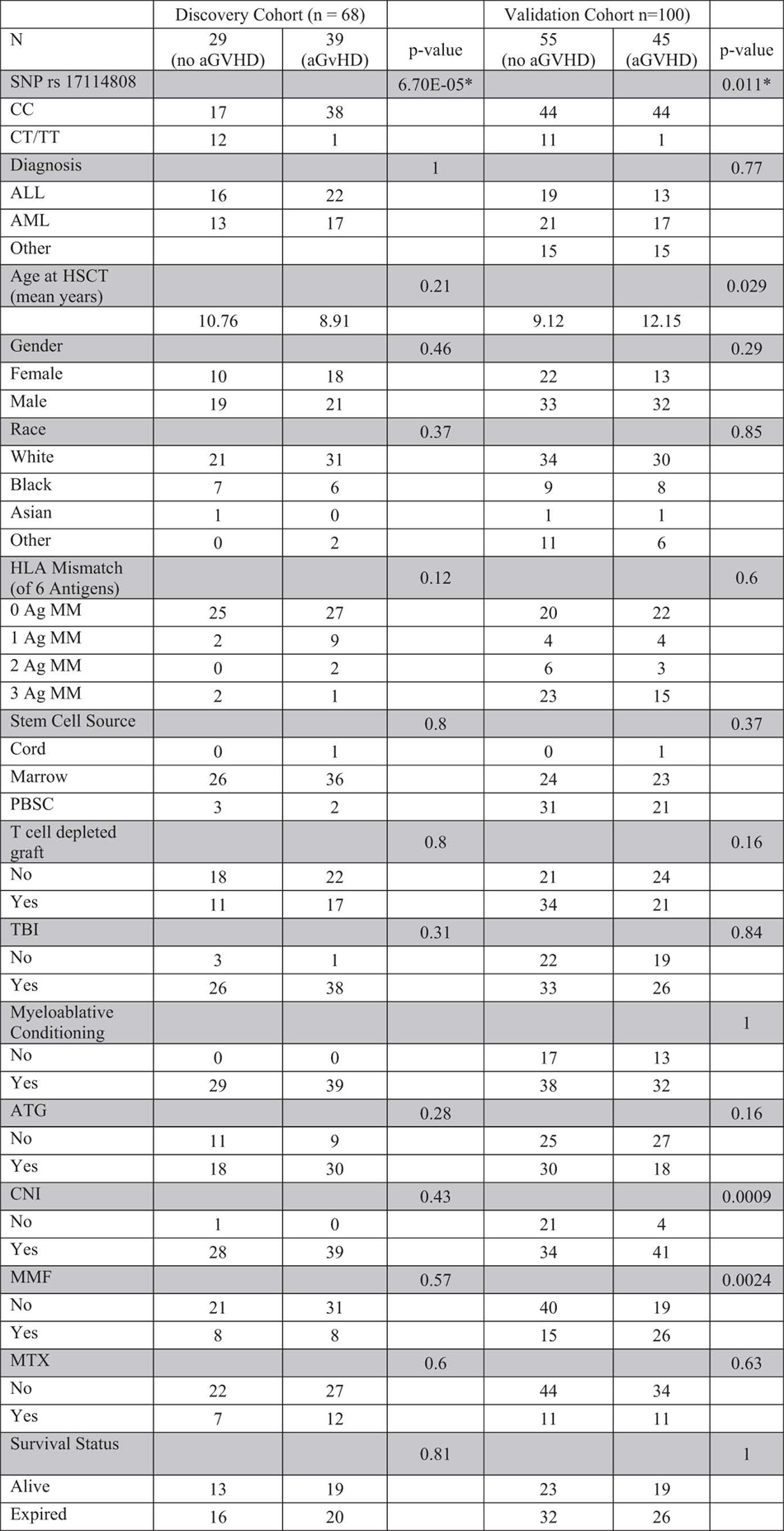
Genotype, patient and transplant characteristics of discovery and validation cohorts according to development of aGVHD.
