# Supplementary material for: Genome-wide single-nucleotide polymorphism analysis revealed SUFU suppression of acute graft-versus-host disease through downregulation of HLA-DR expression in recipient dendritic cells
Source: Sci Rep. 2015 Jun 11;5:11098. doi: 10.1038/srep11098 (PMC4464079; doi:10.1038/srep11098)
Supplement: Supplementary Information [file srep11098-s1.doc]

**Genome-wide single-nucleotide polymorphism analysis revealed SUFU suppression of acute graft-versus-host disease through downregulation of HLA-DR expression in recipient dendritic cells.**

Rafijul Bari,^1,ǁ^ Christine Hartford,^1,ǁ^ Wing Keung Chan,^1^ Queenie Vong,^1^ Ying Li,^1^ Kwan Gan,^1^ Yinmei Zhou,^2^ Cheng Cheng,^2^ Guolian Kang,^2^ Sheila Shurtleff,^3^ Victoria Turner,^3^ Ching-Hon Pui,^3,4,5^ James R. Downing,^3^ Wing Leung,^1,5,*^

**Supplemental materials:**

**Supplemental Figure 1**

**
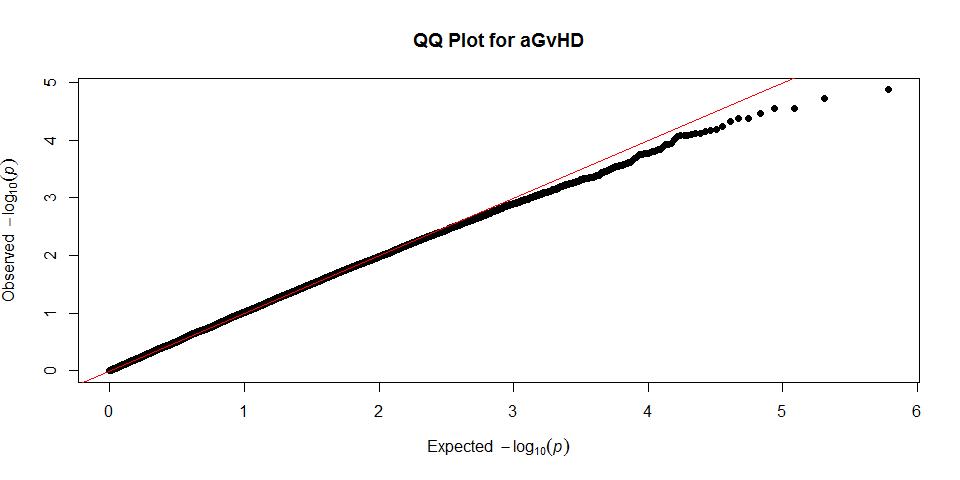
**

**Supplemental Figure 1. Q-Q Plot for genome-wide association results of acute GVHD in the discovery cohort.**

**Supplemental Figure 2**


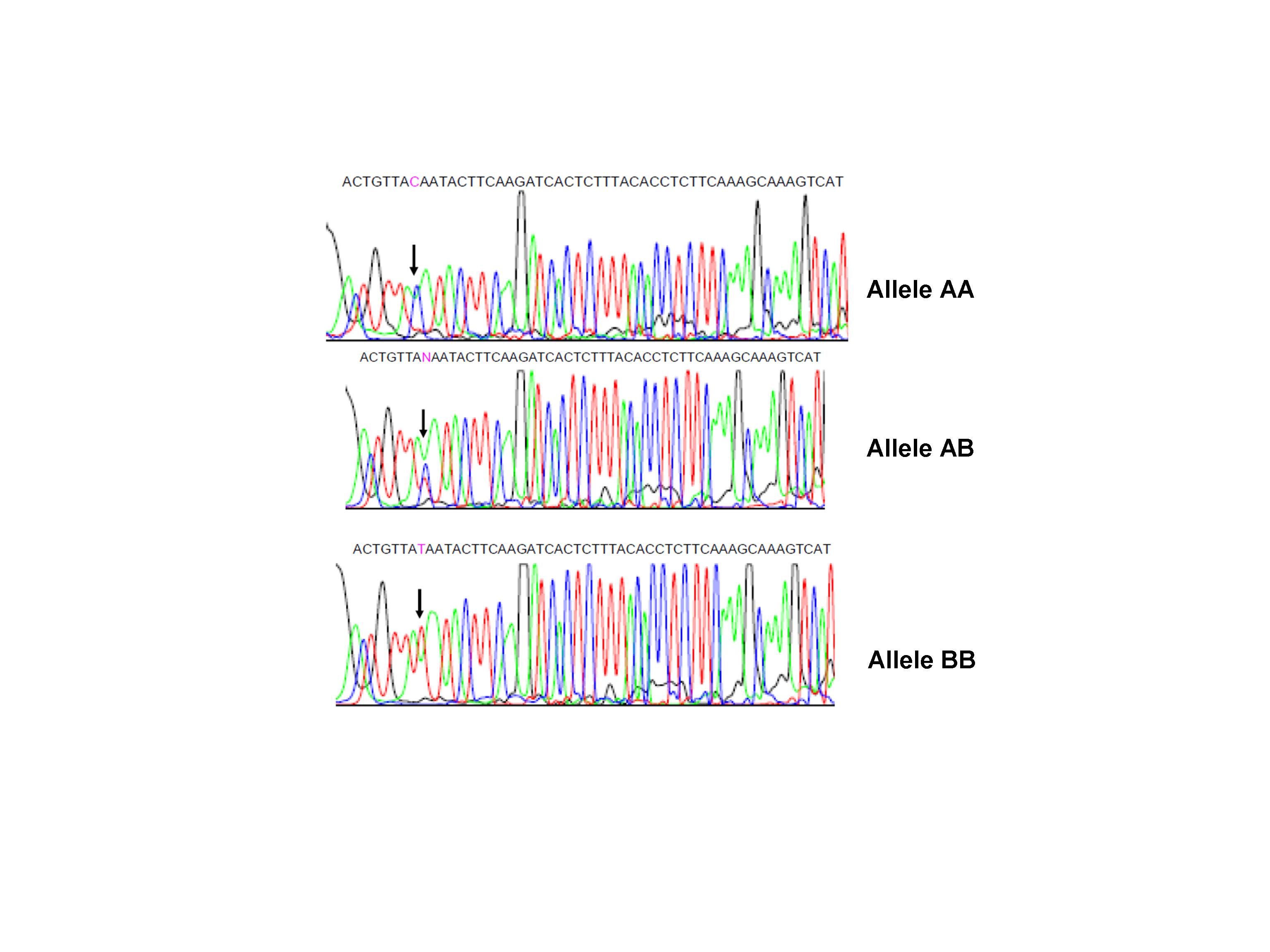


**Allele CC**

**Allele CT**

**Allele TT**

**Supplemental Figure 2. Validation of the SNP assay.** The SNP assay was validated by sequencing the assay products. Purple color and arrows indicate the SNP position.


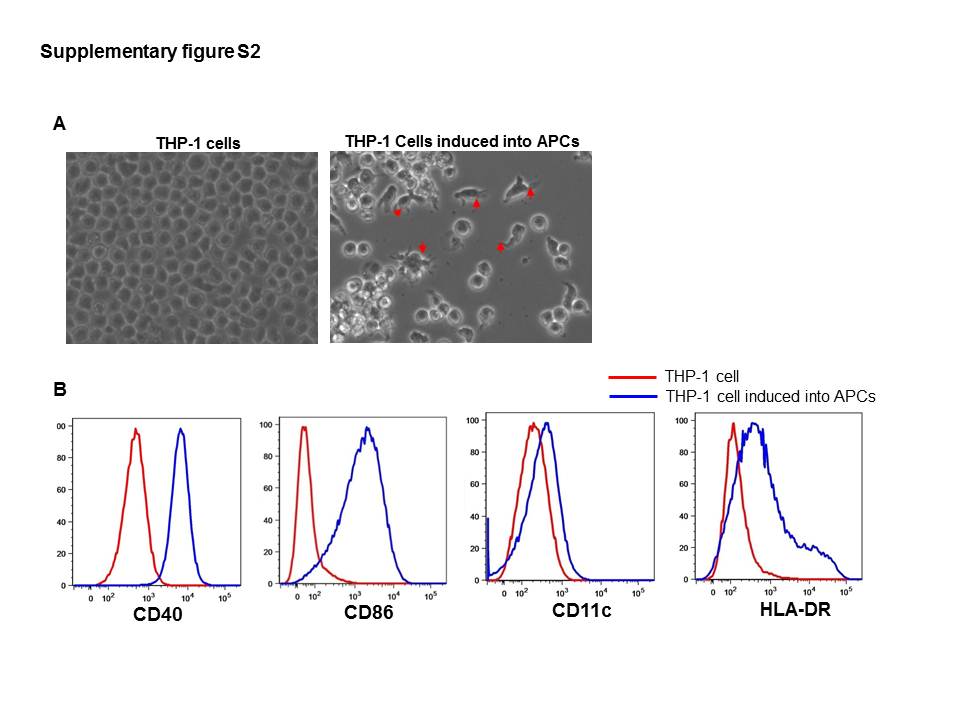
 **Supplemental Figure 3**

**Supplemental Figure 3. Induction of THP-1 cell line into APCs.** A. THP-1 cell line was cultured in RPMI1640 medium (left) or in combination with cytokines that induced them into APCs (right). B. Expression of dendritic cell (DC) markers in THP-1 and THP-1 induced APCs.

**Supplemental Figure 4**


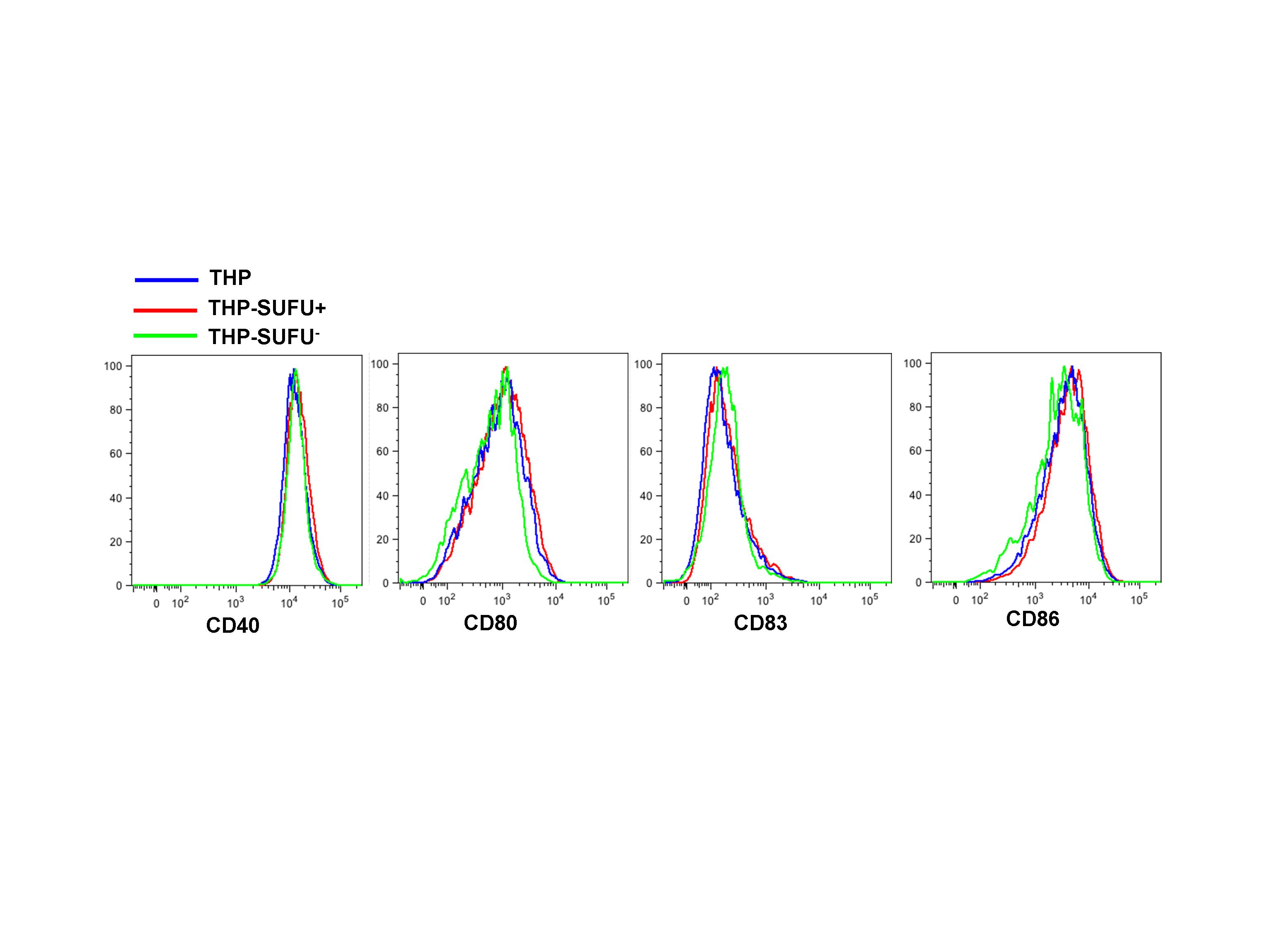


**Supplemental Figure 4. SUFU has no effect on expression of DC markers.** SUFU gene was overexpressed or silenced in THP-1 cell lines followed by inducing them into APCs. Expression of different DC makers was determined by flow cytometric analysis. THP indicates THP-1 cell line; THP-SUFU+ indicates SUFU gene ectopically expressed in THP-1 cell lines; THP-SUFU- indicates SUFU was silenced in THP-1 cell lines by siRNA.

**Supplemental Figure 5**

**
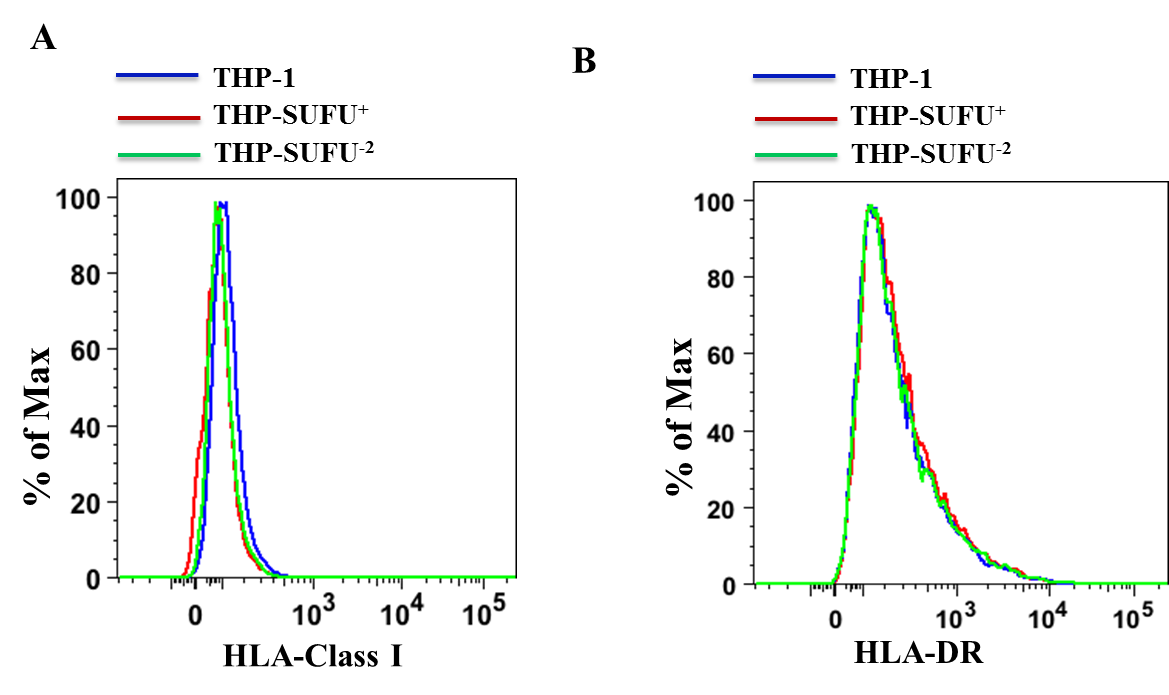
**

**Supplemental Figure 5. SUFU has no effect on expression of HLA-Class I and HLA-DR in THP-1 cells cultured on normal growth medium.** SUFU gene was overexpressed or silenced in THP-1 cell lines. Expression of different HLA-Class I and HLA-DR was determined by flow cytometric analysis. THP indicates THP-1 cell line; THP-SUFU^+^ indicates SUFU gene ectopically expressed in THP-1 cell lines; THP-SUFU^-2^ indicates SUFU was silenced in THP-1 cell lines by siRNA.
